# Neurodevelopmental forecasting in preterms: aEEG at 32 weeks vs. MRI at term in predicting 2-year outcomes

**DOI:** 10.1038/s41372-025-02452-5

**Published:** 2025-10-20

**Authors:** Gina Ancora, Anna Tarocco, Miria Natile, Jasenka Saralija, Alessandra Montesi, Enrico Cocchi

**Affiliations:** 1 Neonatal Intensive Care Unit, Infermi Hospital Rimini, Rimini, Italy; 2 Neonatal Intensive Care Unit, University Hospital S. Anna Ferrara, Ferrara, Italy; 3 Department of Morphology, Surgery and Experimental Medicine, Section of Pathology, Oncology and Experimental Biology, Laboratory for Technologies of Advanced Therapies (LTTA), University of Ferrara, Ferrara, Italy; 4 Childhood And Adolescence Neuropsychiatry Unit, Infermi Hospital Rimini, Rimini, Italy; 5 Department of Medical and Surgical Sciences, Alma Mater Studiorum—University of Bologna, Bologna, Italy; 6 Neonatal and Pediatric Intensive Care Unit, AUSL Romagna, Ravenna, Italy; 7 Department of Precision Medicine and Genomics, Columbia University, New York, USA

**Keywords:** Outcomes research, Neurodevelopmental disorders

## Abstract

**Objective:**

To evaluate the predictive accuracy of amplitude-integrated EEG (aEEG) at 32 weeks corrected age versus brain MRI at term-equivalent age in forecasting neurodevelopmental outcomes in very preterm infants.

**Study design:**

In this prospective cohort study, 71 infants born before 30 weeks’ gestation underwent aEEG at 32 weeks and MRI at term-equivalent age. Neurodevelopmental outcomes were assessed using the Bayley-III at 12 and 24 months, and performance compared through LASSO regression models and ROC analysis.

**Results:**

Burdjalov aEEG and MRI scores were both significantly associated with Bayley-III outcomes. At 12 months, AUCs were 0.93 for aEEG and 0.96 for MRI. At 24 months, AUCs declined but MRI remained superior (0.92 vs. 0.78). The combined model showed the highest accuracy.

**Conclusion:**

aEEG at 32 weeks provides a valuable early indicator of neurodevelopmental outcomes, delivering clinically actionable information weeks before MRI and enabling timely interventions to support brain development in preterm infants.

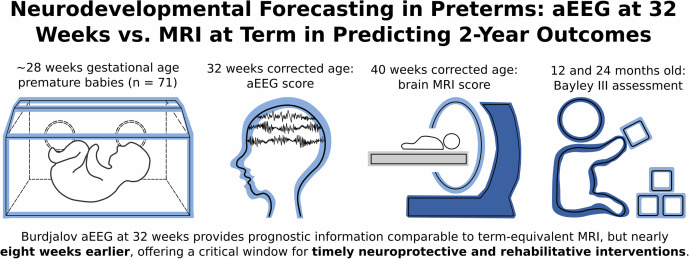

## Introduction

Protecting and promoting brain development is a central objective in neonatal intensive care units (NICUs), particularly as survival rates for preterm infants—especially those born before 30 weeks of gestation—have markedly improved in recent years [1, 2]. Despite these advances, preterm birth remains a major risk factor for neurodevelopmental impairment, including motor, cognitive, and language delays, as well as cerebral palsy and intellectual disability [3, 4]. These outcomes stem not only from overt injuries such as periventricular leukomalacia or intraventricular hemorrhage, but also from more subtle disruptions in brain maturation during a critical window of neurodevelopment spanning 24 to 40 weeks of gestational age [5].

During this period, rapid and complex events shape cerebral connectivity, synaptic development, and myelination. Even in the absence of major lesions, subtle alterations in white or gray matter development may significantly influence long-term neurodevelopmental trajectories [6, 7]. Traditionally, cranial ultrasound has served as the first-line imaging modality during the NICU stay, offering valuable but limited information on subtle white matter injury or brain growth [8, 9]. Magnetic resonance imaging (MRI), particularly when performed at term-equivalent age (TEA), provides greater anatomical detail and has been widely used to assess brain injury severity and to support prognostic counseling [10, 11]. However, the feasibility of routine MRI in all preterm infants is debated, as it requires specialized neuroradiological expertise, logistical coordination, and often sedation—factors that may limit its accessibility and timeliness.

Importantly, structural imaging may not capture all forms of brain dysfunction. Neurodevelopmental impairment may still occur in the absence of major MRI findings, emphasizing the need for additional tools capable of assessing brain function and maturation earlier during NICU hospitalization. In this context, neurophysiological techniques such as conventional EEG (EEG) and amplitude-integrated EEG (aEEG) have garnered increasing attention [12–14]. While EEG provides detailed insight into background activity and seizure detection, its technical complexity and need for expert interpretation limit its widespread use for continuous monitoring in neonatal units. Simplified bedside alternatives like aEEG have become increasingly adopted in modern NICU protocols [15, 16]. aEEG provides a time-compressed, trend-based representation of cerebral electrical activity and is suited for long-term monitoring even in critically ill neonates [17].

When scored using validated systems such as the Burdjalov score—which evaluates continuity, sleep-wake cycling, bandwidth, and lower border amplitude—aEEG offers a semi-quantitative measure of cortical maturation [18, 19]. Although its role in the immediate postnatal period (e.g., first 72 h of life) has been well documented for detecting acute brain injury, its utility at later postmenstrual ages (such as 32 weeks corrected age) for predicting subtle maturational delays remains underexplored.

Recent studies have underscored the value of EEG-based metrics in predicting motor and cognitive outcomes, with some evidence suggesting that delayed functional maturation on aEEG may precede or even predict abnormalities later visualized by TEA-MRI [20]. Nevertheless, comparative data on the prognostic power of aEEG versus MRI—especially when aEEG is performed weeks before MRI—are lacking. Moreover, while MRI is typically used for structural assessment at discharge, aEEG could potentially offer earlier prognostic information that supports more timely implementation of neuroprotective and rehabilitative strategies.

The present study aimed to evaluate the predictive value of aEEG scored with the Burdjalov system at 32 weeks corrected age in a cohort of very preterm infants born before 30 weeks of gestation. Specifically, we sought to: (1) compare the prognostic accuracy of Burdjalov aEEG with TEA-MRI in predicting neurodevelopmental outcomes at 12 and 24 months corrected age; (2) examine the potential additive value of combining both modalities; and (3) explore domain-specific neurodevelopmental trajectories- particularly changes in cognitive, motor and language outcomes over time.

## Methods

### Study design and population

This was a prospective, single-center observational study conducted at the Level III Neonatal Intensive Care Unit (NICU) of Rimini, Italy, between April 1, 2015, and April 1, 2018. This is the follow-up of our previously published study on the same cohort [20]. The study cohort included all inborn and outborn infants born before 30 weeks of gestational age who were consecutively admitted to our institution. The study protocol was approved by the local Ethics Committee (C.E.ROM. protocol number 7457/2015) and written informed parental consent was obtained from all participants. Inclusion criteria were: (1) availability of aEEG performed at 32 weeks of corrected gestational age, (2) brain MRI acquired at term-equivalent age, and (3) completion of standardized neurodevelopmental assessments at both 12 and 24 months corrected age using the Bayley Scales of Infant and Toddler Development, Third Edition (Bayley-III). Infants with major congenital anomalies or chromosomal syndromes were excluded. Infants were also excluded if, at the time of aEEG recording, they exhibited severe clinical conditions contraindicating electrode placement, such as septic or cardiovascular shock. Baseline characteristics collected and their distribution with outcomes under analysis are reported in Table 1. Normality for each variable was assessed through both histogram distribution visual inspection and Shapiro-Wilk test, resulting in non-normal data distribution. Thus, continuous variables are presented as median and interquartile range, while categorical variables are presented as percentages.

**Table 1:** Baseline Perinatal and Clinical Characteristics of the Study Cohort Stratified by Neurodevelopmental Outcome at 12 and 24 Months of Corrected Age.

	Bayley III 12 months			Bayley III 24 months		
	normal *(n* = *51)*	altered *(n* = *20)*	*p*	normal *(n* = *53)*	altered *(n* = *18)*	*p*
sex male	25 (49.0%)	12 (60.0%)	0.6	27 (50.9%)	10 (66.6%)	0.9
gestational age	29 + 1 [27 + 2–29 + 5]	25 + 4 [24 + 1–27 + 4]	<0.001	28 + 7 [27 + 1–29 + 4]	25 + 6 [24 + 0–29 + 1]	0.005
birth weight	1060 [845–1295]	675 [615–877.5]	<0.001	1040 [815–1,270]	822.5 [665–1,002]	0.02
CRIB	7 [5–9]	12 [9.8–13]	<0.001	8 [5–10]	10.5 [8.1–13]	0.02
Apgar 1 min	7 [5.5–8.0]	5 [4–7]	0.02	7 [5–8]	6.5 [4.3–7.8]	0.27
Apgar 5 min	9 [8,9]	8 [7.8–9]	0.1	9 [8,9]	8 [8,9]	0.25
Resuscitation			0.01			0.1
- none	1 (2%)	/		1 (1.9%)	/	
- ventilation	23 (45.1%)	3 (15%)		21 (39.6%)	5 (27.8%)	
- endotracheal tube	17 (33.3%)	5 (25%)		19 (35.8%)	3 (16.7%)	
- chest compression	8 (15.7%)	12 (60.0%)		10 (18.9%)	10 (55.6%)	
- adrenaline	2 (4%)	/		2 (3.6%)	/	
IUGR	6 (12.0%)	5 (26.3%)	0.3	7 (13.5%)	4 (23.5%)	0.6
CS prophylaxis			0.2			0.12
- none	1 (2.0%)	1 (5.0%)		1 (1.9%)	1 (5.6%)	
- incomplete	2 (3.9%)	3 (15.0%)		2 (3.8%)	3 (16.7%)	
- complete	48 (94.1%)	16 (80.0%)		50 (94.3%)	14 (77.8%)	
Prenatal MgSO4			0.6			0.7
- none	24 (47.1%)	11 (55.0%)		26 (49.1%)	9 (50.0%)	
- incomplete	17 (33.3%)	7 (35.0%)		17 (32.1%)	7 (38.9%)	
- complete	10 (19.6%)	2 (10.0%)		10 (18.9%)	2 (11.1%)	
Placenta			0.2			0.2
- normal	21 (42.0%)	4 (20.0%)		22 (42.3%)	3 (16.7%)	
- infection	17 (34.0%)	12 (60.0%)		19 (36.5%)	10 (55.6%)	
- detachment	7 (14.0%)	2 (10.0%)		7 (13.5%)	2 (11.1%)	
- infarction	5 (10.0%)	2 (10.0%)		4 (7.7%)	3 (16.7%)	
pPROM	16 (31.4%)	6 (30.0%)	0.9	16 (30.2%)	6 (33.3%)	0.9
Milk			0.7			0.48
- maternal	35 (68.6%)	13 (65.0%)		36 (67.9%)	12 (66.7%)	
- mix	12 (23.5%)	4 (20.0%)		13 (24.5%)	3 (16.7%)	
- artificial	4 (7.8%)	3 (15.0%)		4 (7.5%)	3 (16.7%)	

This table presents the demographic and perinatal characteristics of the study cohort (71 preterm infants born before 30 weeks of gestation), stratified by neurodevelopmental outcomes assessed with the Bayley-III at 12 and 24 months of corrected age. Given the non-normal distribution of continuous variables, data are reported as medians with interquartile ranges; categorical variables are presented as counts and percentages. Statistical comparisons were conducted using univariate logistic regression. Variables associated with neurodevelopmental outcomes at either time point with a *p*-value < 0.20 were selected for inclusion in the multivariable regression models.

### Neurophysiological assessment: aEEG

aEEG was recorded at 32 weeks post-conceptional age (ranging from 32 + 0/7 to 32 + 6/7) for a minimum duration of 4 h. The 32-week time point was chosen as it marks the completion of neuronal migration and near-resolution of the germinal matrix, coinciding with early cortical plate lamination and rapid synaptogenesis, which together provide a meaningful window to assess functional brain maturation, in line with previous literature [21]. Needle electrodes were positioned at C3-P3 and C4-P4 according to the modified international 10–20 system for neonates. Procedural pain management was conducted according to the Italian national guidelines for neonatal procedural pain [22]. Sedo-analgesic agents administered during monitoring were also documented, as these medications are known to affect neurological activity and aEEG patterns [23–25]. Tracings were acquired using the Brainz Olympic® monitor and independently analyzed in a double-blind fashion by two trained neonatologists. Recordings were scored using the Burdjalov system [18], which evaluates four core parameters of the aEEG tracing: continuity, bandwidth, sleep-wake cycling, and amplitude of the lower border. Each component was scored independently by two trained neonatologists blinded to the clinical outcomes. Discrepancies were resolved by consensus. The overall score, calculated as the sum of the four components, was included in the analysis. The distribution of the Burdjalov score, stratified by neurodevelopmental outcomes, is reported in Table 2.

**Table 2:** MRI findings at 40 weeks post-conceptional age and Burdjalov aEEG score at 32 weeks post-conceptional age in the study cohort, stratified by neurodevelopmental outcome at 12 and 24 months of corrected age.

	Bayley III 12 months			Bayley III 24 months		
	normal *(n* = *51)*	altered *(n* = *20)*	*p*	normal *(n* = *53)*	altered *(n* = *18)*	*p*
MRI findings						
- cystic periventricular leukomalacia	0 (0%)	6 (30.0%)	<0.001	0 (0%)	6 (33.3%)	<0.001
- focal white matter lesions	50 (98.0%)	20 (100%)	0.99	52 (98.1%)	18 (100%)	0.99
- delayed myelination	4 (7.8%)	9 (45.0%)	0.001	4 (7.5%)	9 (50.0%)	<0.001
- thinning of corpus callosum	15 (29.4%)	13 (65.0%)	0.015	17 (32.1%)	11 (61.1%)	0.065
- ventricular dilatation	24 (47.1%)	13 (65.0%)	0.3	28 (52.8%)	9 (50.0%)	0.99
- white matter volume reduction	5 (9.8%)	8 (40.0%)	0.01	7 (13.2%)	6 (33.3%)	0.13
- cortical gray matter abnormalities	46 (90.2%)	18 (90.0%)	1.0	49 (92.5%)	15 (83.3%)	0.35
- delayed gyration	31 (60.8%)	15 (75.0%)	0.45	35 (66.0%)	11 (61.1%)	0.85
- dilated extracerebral CSF spaces	21 (41.2%)	6 (30.0%)	0.51	20 (37.7%)	7 (38.9%)	1.0
- brainstem/cerebellar profile abnormalities	32 (62.7%)	19 (95.0%)	0.02	35 (66.0%)	16 (88.9%)	0.14
- deep gray nuclei abnormalities	13 (25.5%)	18 (90.0%)	<0.001	17 (32.1%)	14 (77.8%)	0.002
- cervical spinal cord signal alteration	30 (58.8%)	17 (85.0%)	0.08	33 (62.3%)	14 (77.8%)	0.4
- cervical spinal cord volume reduction	5 (9.8%)	16 (80.0%)	<0.001	8 (15.1%)	13 (72.2%)	<0.001
Burdjalov score (aEEG)						
Continuity			0.001			0.002
- discontinuous	6 (11.8%)	20 (100%)		7 (13.2%)	9 (50.0%)	
- continuous	45 (88.2%)	10 (50.0%)		46 (86.8%)	9 (50.0%)	
Cycling			0.03			0.06
- waves first appear	0 (0.0%)	2 (10.0%)		0 (0.0%)	2 (11.1%)	
- non definite	10 (19.6%)	8 (40.0%)		12 (22.6%)	6 (33.3%)	
- interrupted	39 (76.5%)	10 (50.0%)		39 (73.6%)	10 (55.6%)	
- non-interrupted	1 (2.0%)	0 (0.0%)		1 (1.9%)	0 (0.0%)	
Amplitude of Lower Border			0.68			0.58
- somewhat depressed	3 (5.9%)	2 (10.0%)		3 (5.7%)	2 (11.1%)	
- elevated	48 (94.1%)	18 (90.0%)		50 (94.3%)	16 (88.9%)	
Bandwidth Span			0.04			0.23
- very immature	1 (2.0%)	2 (10.0%)		2 (3.8%)	1 (5.6%)	
- immature	14 (27.5%)	10 (50.0%)		15 (28.3%)	9 (50.0%)	
- maturing	35 (68.6%)	8 (40.0%)		39 (73.6%)	10 (55.6%)	
Burdjalov Total	10 [9,10]	9 [8–10]	0.14	10 [9,10]	9 [8–10]	0.29

This table reports the distribution of MRI abnormalities and Burdjalov aEEG parameters across infants with normal versus altered neurodevelopmental outcomes, assessed with the Bayley-III at 12 and 24 months. Given the non-normal distribution of continuous variables, data are reported as medians with interquartile ranges; categorical variables are presented as counts and percentages. Statistical comparisons were conducted using univariate logistic regression.

### Neurophysiological assessment: standard EEG

In order to confirm aEEG scoring robustness and to compare the prognostic value of aEEG with EEG, a standard EEG was also recorded at 32 weeks post-conceptional age (32 + 0/7 to 32 + 6/7), generally within one day of the aEEG recording, and repeated at term-equivalent age. Each EEG session lasted at least 30 min. A minimum of eight electrodes (F3, F4, C3, C4, O1, O2, T3, T4) were placed following the neonatal-adapted international 10–20 system. Recordings were acquired using the Micromed® PC system with SystemPlus software. All EEGs were interpreted by a single experienced pediatric neurologist, who rated background activity using a 4-point scale [26, 27]: (1) normal; (2) mildly abnormal—e.g., excess sharp activity, decreased frequency of normal patterns, or prolonged low-voltage periods; (3) moderately abnormal—asymmetry or asynchrony for age, or decreased voltage; (4) severely abnormal—isoelectric or invariant low-voltage activity, burst-suppression, or persistent discontinuity. EEG was used for comparison with the rest of neurophysiological assessment.

### Neuroimaging: brain MRI

Brain MRI was performed at term-equivalent age using a 1.5 T scanner (model: Philips 1.5 Intera). Imaging sequences included T1- and T2-weighted images, diffusion-weighted imaging, and susceptibility-weighted imaging. Image evaluation was based on a structured scoring system adapted from Kidokoro et al. [28], which integrates both qualitative and quantitative assessments of brain abnormalities in preterm infants at term-equivalent age, as detailed in our previous work [20]. Each MRI was scored using a standardized neonatal brain injury scoring system evaluating 12 structural domains: corpus callosum thinning, gyral maturation delay, white matter volume loss, periventricular leukomalacia, ventricular dilatation, deep nuclei abnormalities, extracerebral cerebrospinal fluid enlargement, and cervical spinal cord abnormalities. A total MRI severity score was computed by summing individual domain scores. In accordance with the Italian national guidelines for neonatal procedural pain [22], sedation was achieved via intranasal midazolam at a dose of 0.2–0.3 mg/kg, with repeat dosing administered as needed to ensure adequate immobilization. Radiological assessment was performed by a pediatric neuroradiologist blinded to aEEG, EEG, and outcome data. The distribution of MRI findings across each domain, stratified by neurodevelopmental outcomes, is reported in Table 2.

### Neurodevelopmental outcome assessment

Neurodevelopment was evaluated at 12 and 24 months of age using the Bayley-III score [29]. Cognitive and language scales were administered to each child by examiners annually certified using a video of their administration. The age composite scores were used for statistical analyses. Composite scores were obtained for the cognitive, language (receptive and expressive), motor (fine and gross), socio-emotional, and adaptive behavior domains. Neurodevelopmental outcomes were classified as mild if any individual Bayley-III domain score was <7, severe if any domain was <4, and normal otherwise. For the purpose of binary comparisons, altered outcomes included both mild and severe classifications, contrasted against normal. Changes in Bayley-III scores over time were analyzed both at the overall classification level, composite scores and across individual domains to assess developmental trajectories.

### Statistical analysis

#### Correlation analysis

**Fig. 1:**
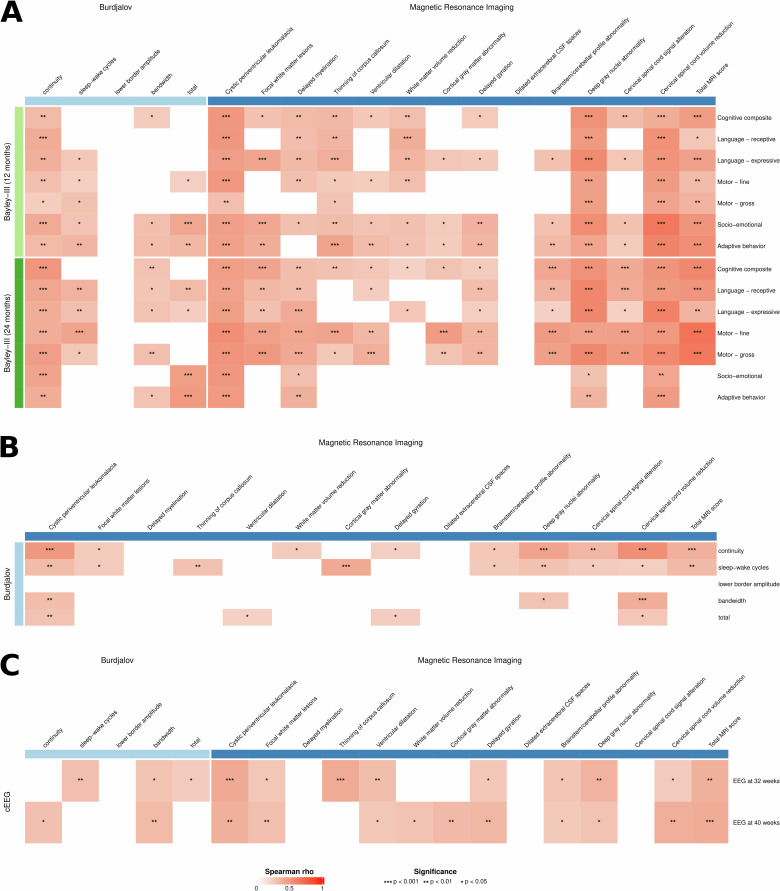
Correlation heatmaps among neurophysiological, neuroimaging, and developmental outcomes. Spearman correlation matrices illustrating relationships among aEEG, MRI, standard EEG, and Bayley-III outcomes. Color intensity reflects correlation strength (Spearman’s rho); only statistically significant correlations (*p* < 0.05) are displayed. **A** Correlation between Burdjalov aEEG scores, brain MRI findings, and Bayley-III composite scores at 12 and 24 months of corrected age. **B** Correlation between Burdjalov aEEG parameters and individual MRI structural domains. **C** Cross-modal correlations among Burdjalov aEEG scores, EEG ratings, and MRI findings.

In order to elucidate neurophysiological, neuroimaging, and neurodevelopment items correlation, we conducted pairwise Spearman rank correlations between aEEG scores, EEG scores, MRI domains, and Bayley-III composite scores at both 12 and 24 months. Separate heatmaps were generated to explore associations: (a) among Burdjalov, MRI, and Bayley-III outcomes, (b) between Burdjalov and MRI components alone, and (c) between Burdjalov-MRI and EEG scores (Fig. 1).

#### Dimensionality reduction and baseline covariate adjustment

Due to high correlation of the demographic and perinatal characteristics, to control for potential confounders while preserving parsimony of the data, we performed a principal component analysis (PCA) on demographic and perinatal variables significantly associated with the outcome in univariate logistic regression (*p* < 0.20). PCA was used to condense highly correlated baseline demographic and perinatal variables (e.g., gestational age, birth weight, illness severity) into a smaller number of independent components. This approach allows us to account for the overall contribution of these baseline characteristics in the predictive models, while avoiding collinearity and overfitting due to the limited sample size. We analyzed PCA results keeping components that explained >80% of the total variance and through visualization cut-off based on the elbow method. The components who survived selection were then served in the regression models to account for baseline characteristics of the sample.

#### Model construction

In order to investigate predictive effectiveness of Burdjalov and MRI scores on neurodevelopmental outcomes at 12 and 24 months, we built four different models using altered vs. normal Bayley III as main outcome:

- Base Model: to account for neonatal characteristics associated (univariate regression *p* < 0.2) with the outcome under analysis.
- Burdjalov Model: base model covariates and Burdjalov items. Among the parameters, LASSO (Least Absolute Shrinkage and Selection Operator) regression was used for variable selection in order to identify most relevant items in shaping the outcome under analysis.
- MRI Model: base model covariates and all MRI domains. Similarly, LASSO selection was applied in order to identify most relevant domains in shaping the outcome.
- Combined Model: Included all selected variables from both the Burdjalov and MRI models, in addition to base model covariates. LASSO selection was applied.

The base model served to investigate the accuracy of neurodevelopmental prediction based on baseline characteristics available soon after birth. The Burdjalov model (available at 32 weeks post-conceptional age), the MRI model (available at 40 weeks post-conceptional age), and the combined model were then developed to test whether functional and/or structural brain assessments could significantly add to these baseline predictors. LASSO regression was applied to identify, among all domains explored in the correlation analysis, those emerging as the strongest predictors of neurodevelopmental outcome within a multivariable framework that accounted for baseline characteristics and other covariates. This approach aimed to determine which Burdjalov and MRI domains carried the highest predictive value on neurodevelopmental outcomes.

#### Model performance evaluation

Receiver operating characteristic (ROC) curves were plotted for each model to evaluate discriminative ability of each model. Area under the curve (AUC) values were calculated with relative 95% confidence intervals. Comparisons among models were performed using the 10,000 bootstrap test for paired ROC curves. The significance threshold was set at *p* < 0.05.

#### Software

All statistical analyses were performed using R (version 4.4.2) with the glmnet, pROC, and corrplot packages.

## Results

### Study population

A total of 107 consecutive eligible preterm infants were referred to our NICU during the study period. Informed written parental consent was not obtained in four cases; two additional infants died before study entry, and two were transferred to another hospital prior to TEA. Furthermore, eight infants were not enrolled due to reduced recruitment activity. No infant was excluded on the basis of clinical instability at the time of aEEG/EEG monitoring. The final study cohort included 91 infants born before 30 weeks of gestation who survived to TEA. Of these, 16 infants did not complete the Bayley-III assessments at 12 and/or 24 months of corrected age, and were therefore excluded from the final analysis. To maximize the accuracy and robustness of the analyses given the limited sample size, we included only complete cases in the final analytic cohort. Accordingly, four additional infants were excluded due to missing data in one or more baseline variables or Bayley-III subscale scores. The remaining 71 preterm infants were included in the analytic cohort; all had complete Burdjalov EEG and aEEG recordings at 32 weeks corrected age, TEA brain MRI and EEG, and neurodevelopmental follow-up with Bayley-III at both 12 and 24 months. Baseline demographic and neonatal clinical characteristics are reported in Table 1. Only three newborns received sedo-analgesic agents or antiseizure medications during aEEG monitoring, and all exhibited normal Burdjalov scores; therefore, this variable could not be included in the analysis. The analysis cohort comprised 37 males (52%), with a median gestational age of 28 weeks [IQR: 26 + 4 to 29 + 3] and a mean birth weight of 950 grams [IQR: 800—1028 g].

### Bayley-III outcome transitions

**Fig. 2:**
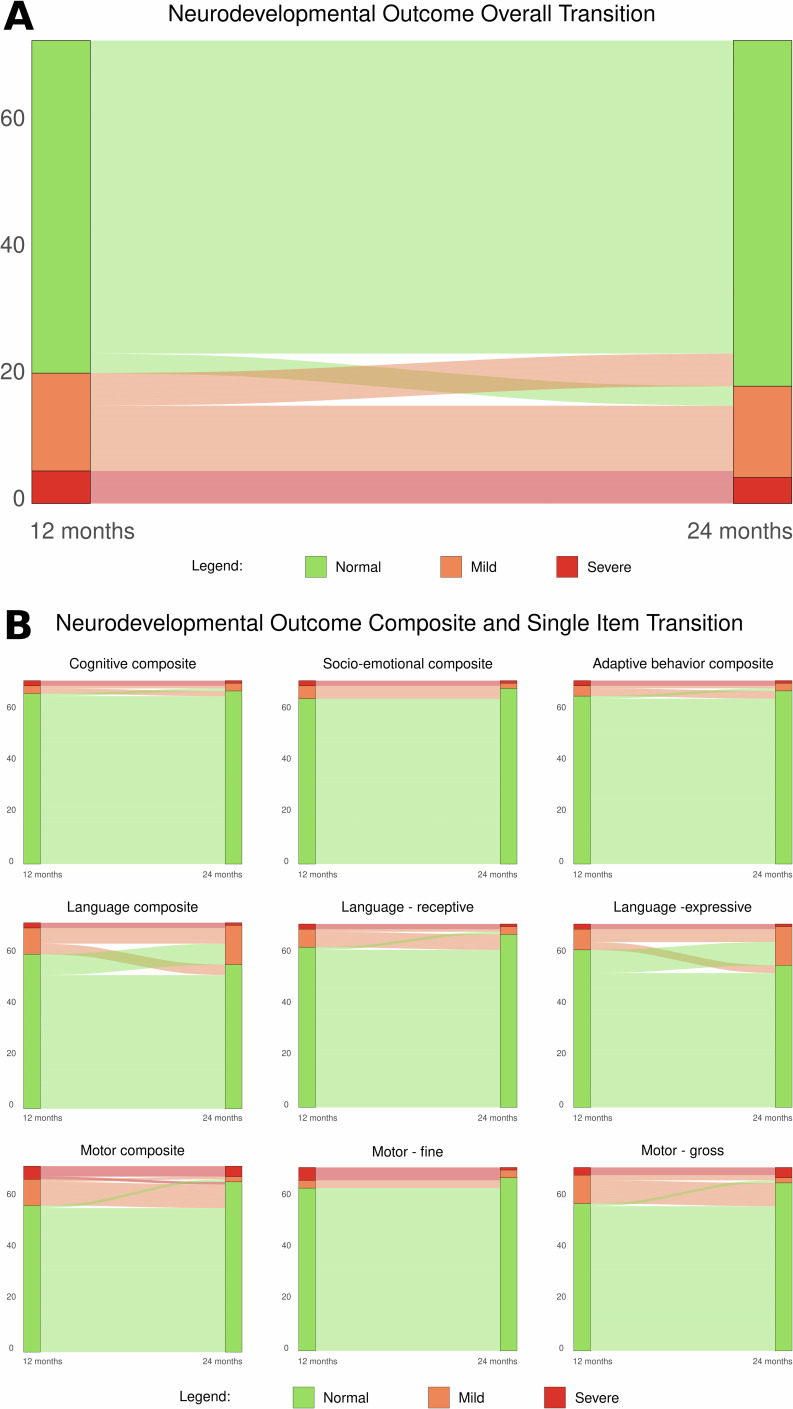
Changes in neurodevelopmental classification and Bayley-III domains between 12 and 24 months. Alluvial plots illustrating transitions in neurodevelopmental classification between 12 and 24 months based on Bayley-III assessments. Outcomes were defined as mild if any individual Bayley-III domain score was <7, severe if <4, and normal otherwise. **A** Overall change in neurodevelopmental classification over time. **B** Domain-specific transitions in cognitive, language, and motor outcomes.

At 12 months of corrected age, 51 infants (71.8%) were classified as having normal neurodevelopment, while 20 (28.2%) exhibited altered development. By 24 months, the proportion of altered cases slightly decreased, with a notable improvement from mild to normal classification in 5 infants, as illustrated in Fig. 2A. Among infants classified as altered at 12 months, deficits were distributed across multiple domains. However, the motor gross domain showed the highest frequency of impairment, followed by expressive language and fine motor domains. By contrast, at 24 months, while the overall rate of abnormal classification declined, the language expressive domain emerged as the most frequently altered, overtaking motor domains. The composite and domain-specific transition analysis (Fig. 2B) confirms these trends: motor gross function was the most frequently improving domain, while expressive language showed the greatest increase in impairment over time.

### Correlation analyses

Heatmap analysis of Spearman correlations (Fig. 1A) showed significant associations between both Burdjalov and MRI scores with Bayley-III composite outcomes at 12 and 24 months:

- Burdjalov: continuity and cycling were significantly correlated with several Bayley III domains at both 12 and 24 months.
- MRI analysis revealed several features that significantly correlated with Bayley-III domains at both 12 and 24 months: thinning of corpus callosum, delayed myelination, cystic periventricular leukomalacia, delayed gyration, deep gray nuclei abnormalities, and cervical spinal cord volume reduction.

The cross-modal correlation heatmap (Fig. 1B) revealed moderate correlations between Burdjalov total score and MRI abnormalities, following the same results identified on our previous work on the matter [20]. Similarly, EEG showed significant correlation with Burdjalov and MRI items at both 32 and TEA (Fig. 1C).

### Predictive models for Bayley-III outcome

#### PCA

The first two principal components (PC1 and PC2), explaining 85.2% of the total variance, were retained. These components represent combined patterns of demographic and perinatal characteristics, and were included as baseline predictors in all subsequent regression models to adjust for underlying clinical differences in the cohort.

#### 12-month outcome

**Fig. 3:**
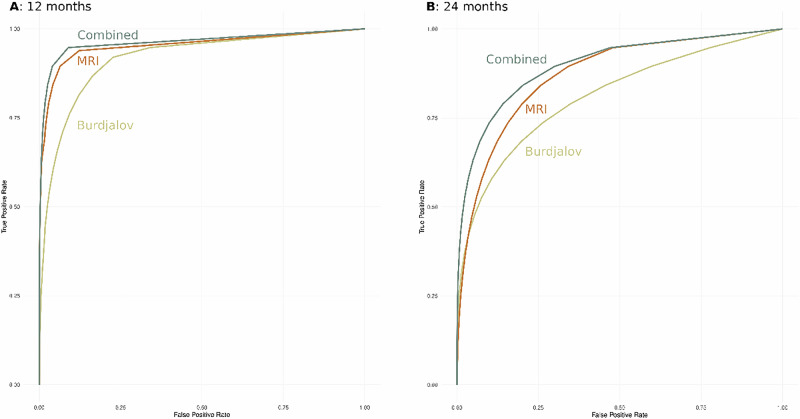
Predictive performance of aEEG and MRI models for Bayley-III outcomes. Receiver Operating Characteristic (ROC) curves evaluating the predictive performance of four models: base model, Burdjalov aEEG model, MRI model, and combined model (Burdjalov + MRI), for Bayley-III outcomes. **A** Prediction of altered outcome at 12 months; **B** Prediction at 24 months.

The performance of the four logistic regression models in predicting altered Bayley-III outcome at 12 months is illustrated in Fig. 3A:

- Base model: AUC = 0.84 (95% CI: 0.73–0.93).
- Burdjalov model: AUC = 0.93 (95% CI: 0.84–0.98).
  - LASSO regression selected: (a) continuity, and (b) cyclicity.
- MRI model: AUC = 0.96 (95% CI: 0.88–0.98).
  - LASSO regression selected: (a) cystic periventricular leukomalacia, (b) thinning of corpus callosum, (c) delayed gyration, and (d) cervical spinal cord volume reduction. Among those, thinning of corpus callosum and cervical spinal cord volume reduction appear as the most consistent predictors.
- Combined model: AUC = 0.97 (95% CI: 0.92–0.99).

No significant difference was detected between the Burdjalov and the MRI model (*p* = 0.4), or between the MRI model and the combined model (*p* = 0.5).

#### 24-month outcome

Model performance at 24 months demonstrated reduced accuracy compared to 12-month outcome, but similar trends (Fig. 3B):

- Base model: AUC = 0.70 (95% CI: 0.55–0.83).
- Burdjalov model: AUC = 0.78 (95% CI: 0.62–0.91); *p* = 0.538 vs. base.
- MRI model: AUC = 0.92 (95% CI: 0.79–0.97); *p* = 0.104 vs. Burdjalov.
- Combined model: AUC = 0.95 (95% CI: 0.87–0.98); *p* = 0.455 vs. MRI.

Similarly to the 12-month outcome, no significant difference was detected between the Burdjalov and the MRI model (*p* = 0.1), or between the MRI model and the combined model (*p* = 0.5), although the difference between the Burdjalov and MRI models increased compared with the 12-month outcome.

## Discussion

Our analysis of neurodevelopmental trajectories in preterm infants born before 30 weeks of gestational age revealed that the majority of these children achieved normal developmental outcomes by 24 months. Notably, we observed a trend toward improvement over time: 14% of infants initially classified as impaired at 12 months demonstrated normalized Bayley-III scores at 24 months. Domain-specific analysis highlighted that motor functions—particularly gross motor skills—were the most frequently impaired at 12 months, yet also showed the greatest potential for recovery. By contrast, language domains, especially expressive language, emerged as the most commonly worsened at 24 months, suggesting a delayed onset/detection of language difficulties in this population. These findings underscore the limitations of relying solely on a single time-point assessment and highlight the need for ongoing neurodevelopmental surveillance, even in infants with reassuring early evaluations.

Our predictive modeling results showed that both the Burdjalov and MRI models achieved high discriminative accuracy at 12 months, with AUC values of 0.93 and 0.96, respectively. At 24 months, while overall model performance declined—as expected given increasing developmental variability—the MRI model seems to outperform the Burdjalov model (AUC 0.92 vs. 0.78, respectively), although no significant difference is reached. Importantly, the combined model consistently outperformed individual models at both time points. This suggests that aEEG and MRI capture partially overlapping but distinct aspects of early brain development: aEEG could more likely reflect functional cortical maturation, while MRI assesses structural integrity and injury patterns.

Burdjalov scoring demonstrated the strongest correlations with language-related domains, while MRI findings were more closely associated with motor and cognitive outcomes, reflecting their respective strengths in capturing functional versus structural brain maturation. The substantial predictive overlap between the two modalities confirms that both are effective in identifying infants at risk for altered neurodevelopmental trajectories—particularly when used in combination. However, a key distinction lies in timing: Burdjalov aEEG can be performed approximately eight weeks earlier than MRI (32 weeks corrected age vs. term-equivalent age), offering a valuable window for initiating early therapeutic interventions. This earlier identification of at-risk infants may enhance the potential for neuroprotective strategies to support more favorable developmental outcomes. In contrast, MRI, while more anatomically informative, often occurs after NICU discharge planning has begun, limiting opportunities for early adjustment in care trajectories.

Moreover, aEEG offers advantages in resource-limited settings where MRI access may be delayed or unavailable [30]. Standardized Burdjalov scoring is feasible with conventional aEEG monitoring equipment and can be integrated into routine NICU neuromonitoring protocols. Its use also avoids the need for sedation and transport required for MRI, which may be contraindicated in critically ill neonates. Furthermore, aEEG, compared to standard EEG, has a simplified electrode setup and can be performed by neonatal intensive care professionals, allowing for continuous and prolonged monitoring of brain electrical activity.

Our findings align with previous work demonstrating the prognostic value of aEEG in preterm populations [31]. Several studies have shown that Burdjalov score can be predictive of later neurodevelopmental outcomes, particularly in infants <32 weeks GA [32]. However, few have directly compared aEEG with MRI using robust statistical modeling. Furthermore, our study builds on our previously published work on the correlation between Burdjalov aEEG and MRI abnormalities at TEA, demonstrating that early functional disturbances are often reflected in later structural findings and can also independently predict neurodevelopmental impairment [20].

### Limitations and future directions

This study has several limitations. First, it was conducted at a single center with a relatively modest sample size, which may limit the generalizability of our findings. Although we used robust modeling strategies and included only complete cases to ensure analytical rigor, other potential sources of bias cannot completely ruled out. Second, although Burdjalov scoring and MRI interpretation were performed by trained, blinded assessors, some degree of subjective variability is inherent to these methods. Additionally, while we included standard EEG recordings for comparative purposes, the study was not powered to evaluate the independent prognostic contribution of standard EEG. A further limitation of this study is the absence of reliable data on major neonatal morbidities such as necrotizing enterocolitis, bronchopulmonary dysplasia, patent ductus arteriosus, and retinopathy of prematurity, which may influence neurodevelopmental trajectories and should be considered in future analyses. Future studies should aim to replicate these findings in larger, multicenter cohorts and explore the added value of advanced quantitative EEG features (e.g., spectral or connectivity analysis) and automated MRI metrics (e.g., volumetric or diffusion-based measures). Long-term follow-up beyond 24 months, including school-age cognitive and behavioral outcomes, is also warranted to assess the durability and specificity of early prognostic markers.

## Conclusions

Burdjalov aEEG performed at 32 weeks corrected age represents a valuable early predictor of neurodevelopmental impairment in very preterm infants. While MRI at term-equivalent age provides more detailed anatomical information and slightly higher predictive accuracy, the earlier availability, feasibility, and functional sensitivity of aEEG make it a highly practical tool for neonatal neuroprognostication. Critically, the ability to identify at-risk infants nearly eight weeks before MRI enables timely implementation of targeted early interventions, potentially improving long-term developmental outcomes in this vulnerable population.

## Data Availability

Completely anonymized data will be available to qualified academic investigators to replicate study results by reasonable request. Data transfer will be regulated by material transfer agreements.
